# Association of neuromuscular reversal by sugammadex and neostigmine with 90-day mortality after non-cardiac surgery

**DOI:** 10.1186/s12871-020-00962-7

**Published:** 2020-02-20

**Authors:** Tak Kyu Oh, Jung-Hee Ryu, Sunwoo Nam, Ah-Young Oh

**Affiliations:** 1 Department of Anesthesiology and Pain Medicine, Seoul National University Bundang Hospital, Seongnam, South Korea; 2 Department of Anesthesiology and Pain Medicine, Seoul National University College of Medicine, Seoul, South Korea

**Keywords:** Muscle relaxation, Mortality, Neostigmine, Sugammadex

## Abstract

**Background:**

Reversing a neuromuscular blockade agent with sugammadex is known to lessen postoperative complications by reducing postoperative residual curarization. However, its effects on 90-day mortality are unknown. Therefore, this study aimed to compare the effects of sugammadex and neostigmine in terms of 90-day mortality after non-cardiac surgery.

**Methods:**

This retrospective cohort study analyzed the medical records of adult patients aged 18 years or older who underwent non-cardiac surgery at a single tertiary care hospital between 2011 and 2016. Propensity score matching and Cox regression analysis were used to investigate the effectiveness of sugammadex and neostigmine in lowering 90-day mortality after non-cardiac surgery.

**Results:**

A total of 65,702 patients were included in the analysis (mean age: 52.3 years, standard deviation: 15.7), and 23,532 of these patients (35.8%) received general surgery. After propensity score matching, 14,179 patients (3906 patients from the sugammadex group and 10,273 patients from the neostigmine group) were included in the final analysis. Cox regression analysis in the propensity score-matched cohort showed that the risk of 90-day mortality was 40% lower in the sugammadex group than in the neostigmine group (hazard ratio: 0.60, 95% confidence interval: 0.37, 0.98; *P* = 0.042). These results were similar in the multivariable Cox regression analysis of the entire cohort (hazard ratio: 0.62, 95% confidence interval: 0.39, 0.96; *P* = 0.036).

**Conclusions:**

This retrospective cohort study suggested that reversing rocuronium with sugammadex might be associated with lower 90-day mortality after non-cardiac surgery compared to neostigmine. However, since this study did not evaluate quantitative neuromuscular function in the postoperative period due to its retrospective design, the results should be interpreted carefully. Future prospective studies with quantitative neuromuscular monitoring in the postoperative period should be performed to confirm these results.

## Background

Neuromuscular blocking agents (NMBAs) have enabled anesthesiologists to achieve optimal surgical conditions, in which patients are immobilized more easily, using smaller amounts of inhaled or intravenous anesthetics [1]. Muscle relaxation with NMBAs has now become part of the classic triad of anesthesia, along with unconsciousness and analgesia [2]. However, like most drugs, NMBAs may cause complications, such as postoperative residual curarization (PORC) [3]. PORC can increase respiratory complications, which may be life-threatening in the immediate postoperative period [4], with an incidence as high as 63.5%, as found by Fortier et al., and 64.7% according to the research by Saager et al. [5, 6].

In 1954, Beecher et al. first reported that the use of NMBAs is associated with anesthesia-related mortality [7]. Since then, PORC has been shown to increase life-threatening critical respiratory events in the immediate postoperative period [8, 9]. In 2017, Bronsert and colleagues reported that PORC, caused by large amounts of NMBAs, may increase 30-day mortality and overall all-cause mortality after non-cardiac surgery [10], in addition to causing critical complications in the immediate postoperative period [4]. This showed that PORC may affect mortality over a relatively long term, as well as in the immediate postoperative period. However, their study primarily used the conventional NMBA reversal agent, neostigmine, and not sugammadex, a newer NMBA reversal agent. Sugammadex is a selective relaxant-binding agent that quickly and effectively reverses the effects of steroidal NMBAs, especially rocuronium and vecuronium [11–13]. Compared with neostigmine, sugammadex more substantially reduces PORC [14, 15], but its effects on postoperative mortality requires further investigation.

Therefore, this study aimed to compare the effects of sugammadex and neostigmine in terms of 90-day mortality after non-cardiac surgery. We hypothesized that using sugammadex for NMBA reversal would improve 90-day mortality after non-cardiac surgery. We thus investigated the 90-day mortality after non-cardiac surgery between sugammadex and neostigmine groups, and also investigated whether the dosage of sugammadex or neostigmine affected the 90-day mortality after non-cardiac surgery.

## Methods

### Design and ethical statement

This retrospective cohort study was approved by the institutional review board (IRB) of the Seoul National University Bundang Hospital (SNUBH) (Approval number: B-1809-495-102, approval date: September 7, 2018). Considering the retrospective nature of the study, in which the medical records of patients who had already completed treatment were analyzed, the requirement for obtaining informed consent was waived by the IRB. This manuscript adheres to the applicable STROBE guidelines.

### Patients

This study analyzed the medical records of adult patients aged 18 years or older who underwent non-cardiac surgery at SNUBH between January 2011 and December 2016. When a patient underwent two or more surgeries, only the final surgery was included in the analysis. Additionally, cases involving incomplete or missing medical records, non-general anesthesia, use of NMBAs other than rocuronium (e.g., cisatracurium), or no NMBA reversal after surgery were excluded from the analysis.

### Rocuronium reversal by sugammadex or neostigmine

SNUBH has generally used rocuronium for general anesthesia in non-cardiac surgeries, while neostigmine or sugammadex has been used as the agent for NMBA reversal after the end of each surgery. There is no strict guideline in our institution to determine the agent (sugammadex or neostigmine) used for NMBA reversal. The decision for using sugammadex or neostigmine is made based on the judgment of the individual anesthesiologist according to the type of surgery, the surgery time, the underlying disease of the patients, and the amount of NMBA used. In most cases, the dosage of sugammadex or neostigmine for NMBA reversal was determined after qualitative (subjective) neuromuscular monitoring using two peripheral nerve stimulators (Innervator 252; Fisher & Paykel Healthcare, New Zealand, and EZStim II, model ES400; Life-Tech International, Stafford, Texas). The residual degree of neuromuscular block from NMBA at emergence was measured after the end of surgery and before extubation. After NBMA reversal administration, the train-of-four (TOF) count was re-checked using the peripheral nerve stimulator to decide on the patient’s readiness for safe extubation. The dosage of sugammadex was determined by depth of neuromuscular block at the end of surgery [16]; 2 mg/kg of sugammadex was administered when the TOF count was ≥1, while 4 mg/kg of sugammadex was administered when the post-tetanic count (PTC) was ≥1. When neostigmine was used, the maximum dose (50 mcg/kg) was administered for NMBA reversal if the TOF count was 1 [16]. If the TOF count was 2–4 at end of surgery, 30–40 mcg/kg was administered for NMBA reversal in the neostigmine group. Additionally, glycopyrrolate was administered with neostigmine to prevent the cholinergic complications of neostigmine. Intraoperative qualitative neuromuscular monitoring was performed throughout the surgery at the discretion of the anesthesiologist. For the present study, we classified patients who were administered sugammadex for NMBA reversal as the sugammadex group and those who were administered neostigmine as the neostigmine group.

### Covariates

Information regarding the patients’ physical characteristics (age [years], sex, body mass index [kg/m^2^]); socioeconomic status (insurance type [National Health Insurance program/Medical Aid Beneficiary program]); marital status (never married/married or living together/divorced or separated/widowed); highest educational attainment (lower than high school/more than or equal to high school, lower than college/more than or equal to college); occupation (office worker/licensed job/house work/self-employed/student, military or laborer/unemployed); and preoperative comorbidities, such as American Society of Anesthesiologists (ASA) physical status, hypertension, diabetes mellitus, ischemic heart disease, cerebrovascular disease, liver disease (fatty liver, liver cirrhosis, and hepatitis), dyslipidemia, chronic kidney disease, and cancer were recorded. Additionally, operative characteristics such as data regarding surgery and anesthesia time (min), emergency surgery, year of surgery, type of non-cardiac surgery (general, thoracic, neuro or spine, orthopedic, plastic, ear-nose-throat, dental, ophthalmic, gynecologic, or urologic surgery), intraoperative rocuronium dosage (mg), and intraoperative qualitative neuromuscular monitoring were collected. The patients in the Medical Aid Beneficiary program are those who are classified as having a low income, and most of their hospital charges are paid by the government. For patients in the National Health Insurance program, approximately two-thirds of hospital charges are covered by the government.

### Ninety-day mortality after surgery

All cases of death within 90 days from the date of surgery were included in the 90-day mortality calculations. We obtained the exact dates of death, including for those patients who were lost to follow-up, from the Ministry of the Interior and Safety in South Korea as of December 31, 2017.

### Statistical analysis

Patient characteristics are presented as mean with standard deviation or number with percentage. First, we performed 1:5 propensity score (PS) matching, a method used to reduce confounding effects in observational studies [17]. The Nearest-Neighbor method, without replacement, was used for PS and the matching caliper was set to 0.2. All covariates were included in the PS model, and logistic regression analysis was performed to calculate PSs, as a logistic model. To determine the balance between the two groups before and after PS matching, the absolute value of the standardized mean difference (ASD) was measured; ASD < 0.2 was defined as well-balanced. After confirming that covariates were well-balanced between the sugammadex and neostigmine groups after PS matching, both Cox regression and logistic regression analyses were performed to investigate the hazard ratio (HR) and odds ratio (OR) with 95% confidence intervals (CIs) for 90-day mortality of the sugammadex group as compared to the neostigmine group in the PS-matched cohort.

Second, we performed uni- and multivariable Cox regression analyses to investigate whether the results from the PS-matched cohort would be generalizable to the entire cohort in our hospital. Despite this, an understanding is required of the association between sugammadex reversal and 90-day mortality with other important confounders, and not only in isolation. All covariates, except for duration of anesthesia were included in the multivariable Cox regression model to avoid multicollinearity with duration of surgery. A log minus log plot was used to determine whether the resulting model satisfied the central assumption of the Cox proportional hazards model. There was no multi-collinearity among all variables in the multivariable model with a variance inflation factor < 2.0. Third, as a secondary sensitivity analysis, we performed multivariable Cox regression analysis to investigate whether the dosage of the two NMBA reversal agents (sugammadex and neostigmine) affected 90-day mortality in each group. The dosage of sugammadex and neostigmine was divided into four groups by quartiles, and all covariates were included in the multivariable Cox regression model (except for duration of surgery). All analyses were performed using the open-source statistical software R (version 3.6.1 with R packages); statistical significance was set at *P* < 0.05.

## Results

### Patients

Non-cardiac surgery was performed in 168,731 cases at our institution between January 2011 and December 2016. Of these, 41,559 cases were initially excluded from analysis as two or more surgeries were performed in one patient during the study period; only the final surgery of each patient was included in the analysis. Next, patients were excluded for the following reasons: 1) incomplete medical records (*n* = 17,120); 2) non-general anesthesia (*n* = 34,675); 3) use of NMBA other than rocuronium (*n* = 5645); and 4) no NMBA reversal after surgery (*n* = 4030). Therefore, 65,702 patients were included in the final analysis: 4578 in the sugammadex group and 61,124 in the neostigmine group. The mean age of the total patients was 52.3 years with a standard deviation of 15.7. Of the total sample group, 23,532 patients (35.8%) received general surgery. The proportion of patients receiving emergency surgery was 0.1% (72/65,702). It was further determined that 34,240 (52.1%) patients were ASA physical status 1 and 29,211 (44.5%) patients were ASA physical status 2.

**Fig. 1:**
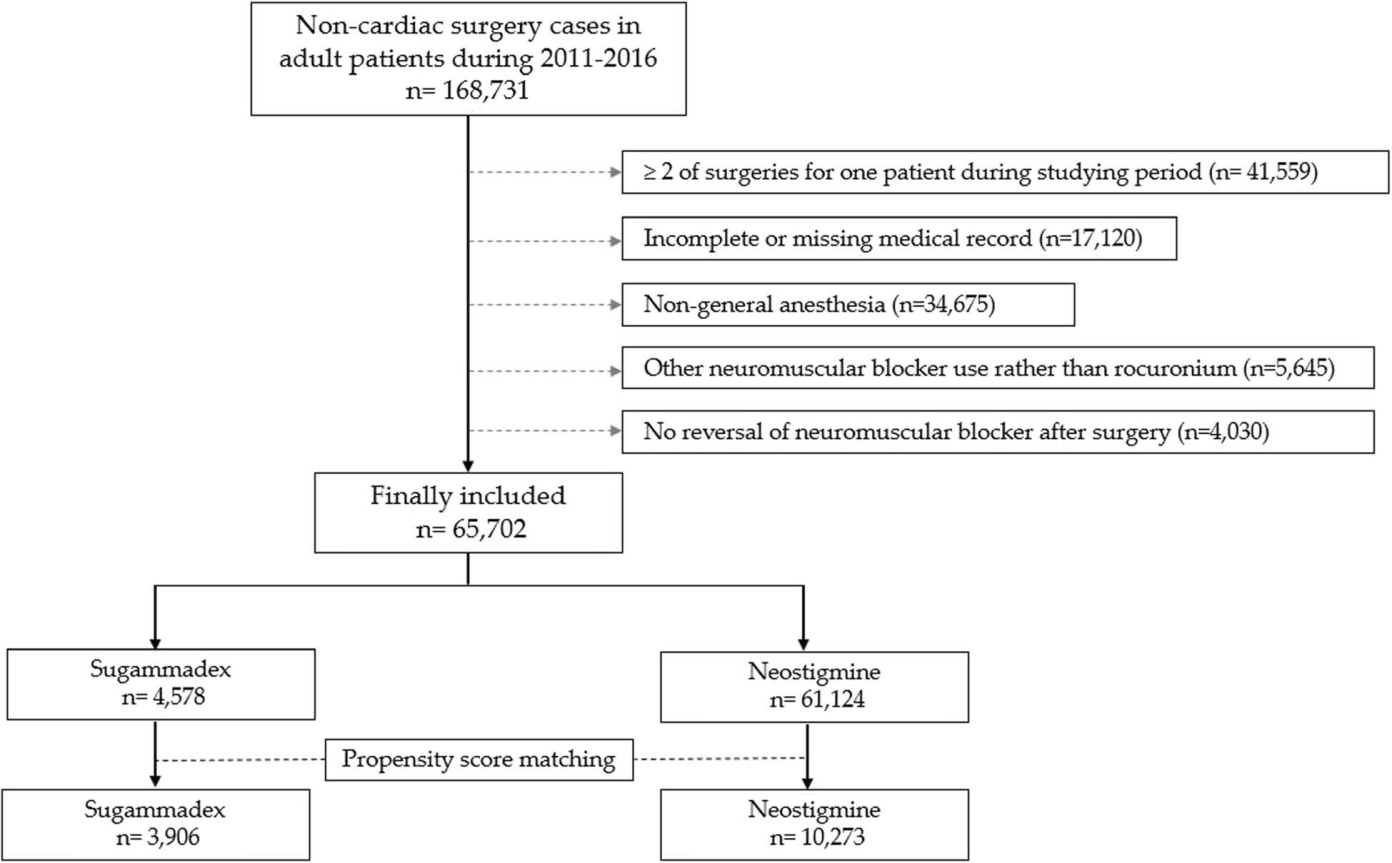
Flow chart of patient selection

After 1:5 PS matching, 3906 and 10,273 patients were isolated in the sugammadex and neostigmine groups, yielding a total of 14,179 patients (Fig. 1). There is a difference between the intended matching ratio (1:5) and actual matching ratio (approximately 1: 2.6) because the matching algorithm was set to nearest neighbor method with a caliper of 0.2. The results of a comparison of the patients’ baseline characteristics before and after PS matching is presented in Table 1. The ASD between the two groups after PS matching was well-balanced (ASD < 0.2; Table 1). Figure S1 show that the PS distribution was similar in the two groups after PS matching. Additionally, the mean sugammadex dosage was 3.24 mg/kg with a standard deviation of 0.72 mg/kg, and the mean neostigmine dosage was 30.9 mcg/kg with a standard deviation of 9.3 mcg/kg.

**Table 1:** Comparison between sugammadex group and neostigmine group before and after propensity score matching

Variables	Before PS matching (*n* = 65,702)			After PS matching (*n* = 14,179)		
	Sugammadex	Neostigmine	ASD	Sugammadex	Neostigmine	ASD
	*n* = 4578	*n* = 61,124		*n* = 3906	*n* = 10,273	
Age, yr	57.7 (14.3)	51.8 (15.7)	0.41	57.2 (14.5)	55.1 (14.9)	0.10
Sex: male	2738 (59.8)	26,517 (43.4)	0.34	2269 (58.1)	5045 (49.1)	0.14
Body mass index, kg m^−2^	24.2 (3.5)	24.0 (3.4)	0.06	24.1 (3.5)	24.0 (3.4)	0.01
Insurance type			0.03			< 0.01
National health insurance program	4488 (98.0)	59,706 (97.7)		3822 (97.8)	10,045 (97.8)	
Medical aid beneficiary program	90 (2.0)	1418 (2.3)		84 (2.2)	228 (2.2)	
Marital status			0.14			0.04
Never married	268 (5.9)	6892 (11.3)		230 (5.9)	784 (7.6)	
Married or living together	3965 (86.6)	49,956 (81.7)		3379 (86.5)	8722 (84.9)	
Divorced or separated	119 (2.6)	1698 (2.8)		104 (2.7)	268 (2.6)	
Widowed	226 (4.9)	2578 (4.2)		193 (4.9)	499 (4.9)	
Highest educational attainment			0.07			0.03
Lower than high school	1066 (23.3)	13,464 (22.0)		903 (23.1)	2219 (21.6)	
More than or equal to high school, lower than college	1434 (31.3)	21,193 (34.7)		1235 (31.6)	3340 (32.5)	
More than or equal to college	2078 (45.4)	26,467 (43.3)		1768 (45.3)	4714 (45.9)	
Occupation			0.19			0.09
Office worker	943 (20.6)	10,838 (17.7)		804 (20.6)	1988 (19.4)	
Licensed job	448 (9.8)	6188 (10.1)		378 (9.7)	1046 (10.2)	
House work	1099 (24.0)	19,753 (32.3)		962 (24.6)	2996 (29.2)	
Self-employed	668 (14.6)	7114 (11.6)		550 (14.1)	1325 (12.9)	
Student, military, or laborer	454 (9.9)	8954 (14.6)		384 (9.8)	1150 (11.2)	
Unemployed	966 (21.1)	8277 (13.5)		828 (21.2)	1768 (17.2)	
Preoperative comorbidities						
ASA physical status			0.20			0.07
1	1891 (41.3)	32,349 (52.9)		1632 (41.8)	4814 (46.9)	
2	2489 (54.4)	26,722 (43.7)		2103 (53.8)	5044 (49.1)	
3	196 (4.3)	2001 (3.3)		169 (4.3)	410 (4.0)	
≥ 4	2 (0.0)	52 (0.1)		2 (0.1)	5 (0.0)	
Hypertension	1299 (28.4)	13,402 (21.9)	0.14	1099 (28.1)	2519 (24.5)	007
Diabetes mellitus	567 (12.4)	5611 (9.2)	0.09	482 (12.3)	1120 (10.9)	0.02
Ischemic heart disease	262 (5.7)	2426 (4.0)	0.08	229 (5.9)	531 (5.2)	0.03
Cerebrovascular disease	205 (4.5)	2130 (3.5)	0.05	182 (4.7)	418 (4.1)	0.04
Liver disease (fatty liver, hepatitis, liver cirrhosis)	168 (3.7)	1405 (2.3)	0.07	142 (3.6)	310 (3.0)	< 0.01
Dyslipidemia	35 (0.8)	596 (1.0)	0.02	32 (0.8)	90 (0.9)	< 0.01
Chronic kidney disease	14 (0.3)	204 (0.3)	< 0.01	14 (0.4)	51 (0.5)	0.01
Cancer	2695 (58.9)	18,651 (30.5)	0.58	2242 (57.4)	5080 (49.5)	< 0.01
Operative Characteristics						
Surgery time, min	150.7 (95.1)	111.8 (145.6)	0.39	148.6 (99.4)	131.4 (102.2)	0.05
Anesthesia time, min	190.8 (104.8)	150.7 (95.1)	0.38	188.3 (105.4)	169.6 (109.0)	0.04
Emergency surgery	3 (0.1)	69 (0.1)	0.02	2 (0.1)	8 (0.1)	0.01
Type of non-cardiac surgery			1.23			0.08
General surgery	3185 (69.6)	20,347 (33.3)		2777 (71.1)	5889 (57.3)	
Thoracic surgery	40 (0.9)	2974 (4.9)		40 (1.0)	214 (2.1)	
Neuro or spine surgery	81 (1.8)	6552 (10.7)		81 (2.1)	461 (4.5)	
Orthopedic surgery	52 (1.1)	8654 (14.2)		52 (1.3)	440 (4.3)	
Plastic, ENT, Dental, Eye surgery	102 (2.2)	10,458 (17.1)		128 (3.0)	639 (6.2)	
Gynecologic or Urologic surgery	1128 (24.5)	12,139 (19.9)		838 (21.5)	2630 (25.6)	
Intraoperative rocuronium dosage, mg			1.26			0.02
< 50	3 (0.1)	130 (0.2)		3 (0.1)	11 (0.1)	
50–100	1082 (23.6)	47,049 (77.0)		1080 (27.6)	5145 (50.1)	
> 100	3493 (76.3)	13,945 (22.8)		2823 (72.3)	5117 (49.8)	
Intraoperative qualitative neuromuscular monitoring	78 (1.7)	1.199 (2.0)	0.02	57 (1.5)	128 (1.2)	0.01
Year of surgery			1.83			0.09
2011–2012	0 (0.0)	22,198 (36.3)		0 (0.0)	776 (7.6)	
2013–2014	475 (10.4)	18,215 (29.8)		475 (12.2)	2156 (21.0)	
2015–2016	4103 (89.6)	20,711 (33.9)		3431 (87.8)	7341 (71.5)	

Presented as number (percentage) or mean (standard deviation)

*PS* propensity score; *ASD* Absolute value of standardized mean difference; *ASA* American Society of Anesthesiologists; *TOF* train-of-four

### Ninety-day mortality after non-cardiac surgery

The results of survival analysis for 90-day mortality before and after PS matching are presented in Table 2. The 90-day mortality in the sugammadex and neostigmine group was 0.6% (26/4578) and 0.6% (365/61,124), respectively, before PS matching in the entire cohort. The Cox regression and logistic regression models showed no statistically significant difference in 90-day mortality between the two groups in the entire cohort (*P* = 0.806 and *P* = 0.804, respectively). However, after PS matching, the 90-day mortality in the sugammadex group and neostigmine group was 0.5% (20/3906) and 0.8% (87/10,273), respectively. The Cox regression and logistic regression models showed that use of sugammadex was associated with a 40% lower 90-day mortality risk than use of neostigmine (HR: 0.60, 95% CI: 0.37, 0.98; *P* = 0.042 and OR: 0.60, 95% CI: 0.37, 0.98; *P* = 0.042). In the multivariable Cox regression model in entire cohort, use of sugammadex was also associated with 38% lower 90-day mortality risk than use of neostigmine (HR: 0.62, 95% CI: 0.39, 0.96; *P* = 0.036; Table 3).

**Table 2:** Survival analysis for 90-day mortality before and after propensity score matching

Model	Event (%)	Cox regression analysis		Logistic regression analysis	
		Hazard ratio (95% CI)	*P*-value	Odds ratio (95% CI)	*P*-value
Before propensity score matching					
Neostigmine group	365 / 61,124 (0.6%)	1			
Sugammadex group	26 / 4578 (0.6%)	0.95 (0.64, 1.42)	0.806	0.95 (0.64, 1.42)	0.804
After propensity score matching					
Neostigmine group	87/10,273 (0.8%)	1		1	
Sugammadex group	20/3906 (0.5%)	0.60 (0.37, 0.98)	0.042	0.60 (0.37, 0.98)	0.042

*CI* confidence interval

**Table 3:** Univariable and multivariable Cox regression model for 90-day mortality in entire cohorts

Model	Univariable model		Multivariable model^a^	
	Hazard ratio (95% CI)	*P*-value	Hazard ratio (95% CI)	*P*-value
Age, yr	1.07 (1.06, 1.08)	< 0.001	1.03 (1.02, 1.04)	< 0.001
Sex: male (vs female)	2.34 (1.90, 2.88)	< 0.001	1.81 (1.34, 2.43)	< 0.001
Body mass index, kg m^−2^	0.80 (0.78, 0.83)	< 0.001	0.83 (0.80, 0.85)	< 0.001
Insurance type: Medical aid beneficiary program	2.93 (1.95, 4.39)	< 0.001	1.30 (0.85, 1.98)	0.229
Marital status				
Never married	1	(< 0.001)	1	(0.191)
Married or living together	1.80 (1.19, 2.73)	0.005	0.60 (0.37, 0.97)	0.038
Divorced or separated	1.31 (0.59, 2.92)	0.504	0.52 (023, 1.19)	0.119
Widowed	3.63 (2.16, 6.13)	< 0.001	0.56 (0.31, 1.04)	0.065
Highest educational attainment				
Lower than high school	1	(< 0.001)	1	(0.056)
More than or equal to high school, lower than college	0.42 (0.33, 0.54)	< 0.001	0.79 (0.61, 1.02)	0.065
More than or equal to college	0.35 (0.28, 0.45)	< 0.001	0.75 (0.58, 0.97)	0.027
Occupation				
Office worker	1	(< 0.001)	1	(0.076)
Licensed job	0.84 (0.46, 1.51)	0.553	0.88 (0.49, 1.60)	0.677
House work	1.36 (0.91, 2.03)	0.129	1.19 (0.74, 1.91)	0.467
Self-employed	1.43 (0.88, 2.31)	0.150	0.91 (0.56, 1.49)	0.716
Student, military, or labourer	1.81 (1.67, 2.80)	0.008	1.07 (0.68, 1.68)	0.778
Unemployed	6.73 (4.66, 7.91)	< 0.001	1.47 (0.98, 2.21)	0.063
ASA physical status				
1	1	(< 0.001)	1	(< 0.001)
2	7.73 (5.42, 11.03)	< 0.001	5.38 (3.69, 7.91)	< 0.001
3	54.56 (37.43, 79.53)	< 0.001	21.56 (14.02, 33.16)	< 0.001
≥ 4	138.51 (61.52, 311.82)	< 0.001	42.44 (17.89, 100.66)	< 0.001
Hypertension	1.71 (1.39, 2.11)	< 0.001	0.66 (0.52, 0.83)	< 0.001
Diabetes mellitus	2.29 (1.78, 2.95)	< 0.001	0.97 (0.75, 1.27)	0.824
Ischemic heart disease	3.87 (2.91, 5.14)	< 0.001	0.90 (0.66, 1.23)	0.509
Cerebrovascular disease	3.02 (2.17, 4.21)	< 0.001	0.90 (0.64, 1.27)	0.546
Liver disease (fatty liver, hepatitis, liver cirrhosis)	2.56 (1.68, 3.90)	< 0.001	1.81 (1.18, 2.76)	0.006
Dyslipidemia	3.28 (1.85, 5.82)	< 0.001	1.20 (0.67, 2.15)	0.540
Chronic kidney disease	8.95 (4.91, 16.30)	< 0.001	1.89 (1.02, 3.52)	0.045
Cancer	5.27 (4.23, 6.56)	< 0.001	3.32 (2.64, 4.17)	< 0.001
Surgery time, hour	1.01 (1.01, 1.02)	< 0.001	1.01 (1.01, 1.02)	< 0.001
Anesthesia time, hour	1.27 (1.22, 1.32)	< 0.001		
Emergency surgery	2.36 (0.33, 16.78)	0.392	1.32 (0.20, 10.28)	0.727
Type of non-cardiac surgery				
General surgery	1	(< 0.001)	1	(0.007)
Thoracic surgery	2.14 (1.53, 2.99)	< 0.001	1.36 (0.97, 1.92)	0.079
Neuro or spine surgery	1.04 (0.75, 1.43)	0.832	1.99 (1.38, 2.85)	< 0.001
Orthopedic surgery	0.65 (0.46, 0.93)	0.017	1.41 (0.97, 2.04)	0.074
Plastic, Ear-nose-throat, Dental, Eye surgery	0.54 (0.38, 0.77)	0.001	1.31 (0.91, 1.88)	0.150
Gynecologic or Urologic surgery	0.67 (0.50, 0.90)	0.008	1.04 (0.77, 1.42)	0.785
Year of surgery				
2011–2012	1	(0.238)	1	(0.076)
2013–2014	0.95 (0.74, 1.21)	0.672	0.94 (0.73, 1.21)	0.622
2015–2016	0.82 (0.65, 1.04)	0.099	0.75 (0.58, 0.97)	0.029
Intraoperative qualitative neuromuscular monitoring	0.05 (0.00, 1.19)	0.064	0.00 (0.00-)	0.893
Intraoperative Rocuronium dosage, per 1 mg kg^−1^	1.54 (1.39, 1.70)	< 0.001	1.00 (0.97, 1.03)	0.772
Reversal by Sugammadex (vs neostigmine)	0.95 (0.64, 1.42)	0.806	0.62 (0.39, 0.96)	0.036

^a^ All covariates were included in multivariable model, except for surgery time to avoid multi-collinearity in multivariable Cox regression model

*CI* confidence interval; *ASA* American Society of Anesthesiologists; *TOF* train-of-four

### Subgroup analysis according to dosage of sugammadex and neostigmine

Table 4 shows the results of subgroup analysis for 90-day mortality according to dosage of neostigmine and sugammadex. In the multivariable Cox regression model in the neostigmine group, when compared to the Q1 group, the Q2, Q3 and Q4 groups were not associated with increased 90-day mortality (all *P* > 0.05). In the multivariable Cox regression model in the sugammadex group, when compared to the Q1 group, the Q2, Q3 and Q4 groups were not associated with increased 90-day mortality (all *P* > 0.05).

**Table 4:** Subgroup analysis for 90-day mortality according to dosage of neostigmine and sugammadex

Variable	Multivariable Cox regression model	*P*-value
	Hazard ratio (95% CI)	
Neostigmine dosage, mcg kg^− 1^ (*n* = 61,124)		
Q1 < 27.5 (*n* = 15,108)	1	
27.5 ≤ Q2 < 31.8 (*n* = 15,464)	0.75 (0.53, 1.06)	0.102
31.8 ≤ Q3 < 36.4 (*n* = 15,582)	0.89 (0.63, 1.25)	0.491
36.4 ≤ Q4 (*n* = 14,970)	0.96 (0.66, 1.42)	0.853
Sugammadex dosage, mg kg^−1^ (*n* = 4578)		
Q1 < 2.8 (*n* = 1214)	1	
2.8 ≤ Q2 < 3.1 (*n* = 963)	0.94 (0.11, 7.78)	0.955
3.1 ≤ Q3 < 3.6 (*n* = 1278)	2.27 (0.34, 15.15)	0.397
3.6 ≤ Q4 (*n* = 1123)	2.91 (0.30, 27.91)	0.354

All covariates were included in the multivariable model

*CI* confidence interval

## Discussion

The results of this retrospective cohort study suggested that reversing rocuronium with sugammadex is associated with a lower 90-day mortality rate after non-cardiac surgery when compared with neostigmine. This association was statistically significant in the PS-matched cohort, but not in the entire cohort. Additionally, the dosage of sugammadex or neostigmine in both groups was not associated with 90-day mortality in subgroup analyses.

The 90-day mortality rate (0.6%) after surgery was relatively lower in this study than that reported in a previous study (4% hospital mortality after surgery) [18]. This difference might be caused by the characteristics of the surgical population of our study. We excluded relatively high-risk patients who underwent cardiac surgery, and patients who did not receive NMBA reversal in order to receive mechanical ventilation in the intensive care unit (ICU) during the immediate postoperative period. Additionally, the patients with end-stage renal disease might have been excluded from this study, because they usually received atracurium or cisatracurium, rather than rocuronium, during surgery.

When interpreting the present findings, consideration must be given to the fact that both quantitative and qualitative neuromuscular function monitoring was not routine practice, it was performed at the discretion of the attending anesthesiologists. Hence, our data reflects the results of our everyday clinical practice rather than standardized best practice. Previous surveys also have shown the limited use of a neuromuscular monitoring, the majority of which comprised qualitative monitoring, while the use of quantitative monitoring was far less common [19–21]. In this study, we measured the TOF count after surgery at emergence using qualitative rather than quantitative neuromuscular monitoring, because quantitative neuromuscular monitoring at the end of surgery was not available during the study period (2011–2016). Given that quantitative and objective neuromuscular monitoring is considered the gold standard to detect PORC [3, 22], the use of qualitative and subjective neuromuscular monitoring may have affected the results of this study. Although we did not have access to postoperative TOF ratio data, we assume that PORC would have been more frequently associated with neostigmine compared to sugammadex for several reasons. The ability of neostigmine to reverse neuromuscular blockade is limited. Even when the full recommended dose of 70 mcg/kg is administered at a TOF count of 4, the recovery has been shown to be unsatisfactory [23, 24]. Additionally, neostigmine overdose in fully recovered patients could result in a reduction of muscle strength.

The contribution of PORC to increased postoperative mortality and morbidity has been well-documented in the literature. In a large retrospective study in 2005, Arbous and colleagues reported that inadequate NMBA reversal is an independent risk factor for increased 24-h postoperative mortality and morbidity [25]. Furthermore, Murphy and colleagues reported that residual block increases life-threatening, critical respiratory events in the postoperative recovery room [9]. Bronsert and colleagues were the first to show that PORC caused by NMBA may affect complications in the immediate postoperative period, as well as long-term mortality after non-cardiac surgery [10]. However, they used data from five Veterans Health Administration hospitals in the United States, from 2003 to 2008; sugammadex was only approved by the Food and Drug Administration in the United States in December 2015. Thus, Bronsert and colleagues were unable to consider the effect of sugammadex. In contrast, our institution (SNUBH) has consistently used sugammadex as a drug for NMBA reversal since 2011. Thus, our study presents novel data in that we have described the effects of sugammadex on 90-day mortality after non-cardiac surgery.

A recently published study on the post-anesthesia pulmonary complications after use of muscle relaxants (POPULAR) trial reported that use of NMBA in general anesthesia is associated with a risk of postoperative pulmonary complications (PPCs), while NMBA reversal with sugammadex was not significantly associated with PPCs [26]. The results of the POPULAR trial differed from those in our study, and the difference could be explained by a human factor of the anesthetist, because approximately one-third of patients who underwent NMBA monitoring were extubated at a TOF ratio < 0.9. In this context, reversal of sugammadex was independently associated with a better safety profile. Additionally, two recent meta-analyses concluded that sugammadex not only reversed residual NMBA more rapidly and completely by encapsulation than did the anticholinesterase reversal drug (neostigmine), but it was also associated with a better safety profile, specifically regarding residual NMBA after reversal [27, 28]. The meta-analysis reported that reversal by sugammadex might lead to a lower incidence of residual NMBA, and related side-effects, than reversal by neostigmine.

Some plausible mechanisms have been suggested to explain why PORC could result in an increase of various complications beyond the immediate postoperative period [29]. First, PORC and NMBA reversal with high-dose neostigmine is known to be associated with development of hypopharyngeal weakness [30, 31], which could lead to a risk of aspiration and pneumonia, and overall higher rates of PPCs [26, 32, 33]. Furthermore, we recently published an observational study in which we showed that NMBA reversal by sugammadex was associated with lower 30-day unplanned readmission rates, hospital charges, and length of hospital stay after major abdominal surgery [34]. In this study, we showed that reversal by sugammadex, which might be closely related to reduced PORC, could have relatively long-term effects on outcome, beyond the immediate postoperative period. Second, NMBA reversal with neostigmine and anticholinesterases might be associated with increased risk of cardiovascular complications in the high-risk group (age > 70 years, undergoing vascular surgery, prior history of atrial fibrillation) [29, 35]. Furthermore, a recent cohort study reported that PORC in the post-anesthesia care unit is associated with higher rates of ICU admission [36]. Therefore, the results of our current study suggested that efforts to minimize PORC using sugammadex could result in improvement of fatal outcomes, such as 90-day mortality.

The results of subgroup analyses regarding dosage of neostigmine and sugammadex were also notable in this study. Although the results of the main analysis showed that sugammadex might be potentially beneficial in lowering 90-day mortality, the results of subgroup analyses showed that the dosage of sugammadex or neostigmine did not affect 90-day mortality in either group. In our institution, the dosage of neostigmine (30–50 mcg/kg) or sugammadex (2–4 mg/kg) was determined following the surgical procedure using a peripheral nerve stimulator, in most cases. These results suggested that although the sugammadex group was associated with a lower 90-day mortality than the neostigmine group, the dosage of the agent received in both groups did not affect the 90-day mortality. This may be because the TOF count was checked after surgery in most cases in order to determine the required dosage of neostigmine or sugammadex.

It is possible that the dosage of the agent received in the neostigmine and sugammadex groups affected the results of the main analysis. 2 mg/kg of sugammadex was administered when the TOF count was ≥1, and 4 mg/kg of sugammadex was administered when the PTC was ≥1. In the neostigmine group, the maximum dose of neostigmine (50 mcg/kg) was used for NMBA reversal if the TOF count was 1 (i.e. a moderate block) at the end of surgery in this study. While doses of sugammadex lower than 2.0 mg/kg can be sufficient to reverse residual rocuronium-induced neuromuscular block with a TOF ratio of 0.2, even 70 mcg/kg of neostigmine cannot reliably reverse a residual neuromuscular block with a TOF ratio of 0.2 within 10 min [37]. This suggests that the administration of 50 mcg/kg of neostigmine in this study might have been an insufficient dose to reverse a block with a TOF count of 1 without quantitative monitoring [23, 24]. Therefore, the variation of doses in the sugammadex and the neostigmine groups may not be equipotent, and in the absence of quantitative monitoring, it is possible that the neostigmine group in this study was reversed and extubated using a suboptimal dose of neostigmine.

Our study had a few limitations. First, due to the retrospective nature of the study, the quality and accuracy of the data may not meet the standard required for prospective studies. Second, this is a single center study, which limits the generalizability of the findings. Third, the sugammadex group comprised only 7.0% of the entire cohort; thus, the sample size was substantially reduced after propensity score matching. Fourth, we did not evaluate the PORC at PACU admission in this study, which could limit the results of this study. Fifth, we could not provide information regarding the patients who suffered from PPCs, due to the retrospective nature of the study. Additionally, we did not provide information regarding the causes of 90-day mortality in this study. Lastly, quantitative neuromuscular monitoring was not performed in the study period at emergence after surgery to determine the type or dosage of NMBA reversal agent. Only qualitative neuromuscular monitoring was performed; however, qualitative monitoring-guided neostigmine reversal is no longer recommended due to the risk of PORC [3, 22, 38]. Therefore, our results might have differed if quantitative monitoring was performed to guide the dosage of neostigmine; further study is needed to confirm this.

## Conclusions

This retrospective study showed that the use of sugammadex for rocuronium reversal is associated with a reduced 90-day mortality after non-cardiac surgery. However, since this study did not evaluate quantitative neuromuscular function in the postoperative period due to its retrospective design, the results should be interpreted carefully. Future prospective studies with quantitative neuromuscular monitoring in the postoperative period should be performed to confirm this association.

## Supplementary information

**Additional file 1 Figure S1.** Distribution of propensity scores before and after propensity score matching

## Data Availability

The datasets generated and analyzed during the current study are available from the corresponding author on reasonable request.

## Abbreviations

ASD: Absolute value of the standardized mean difference
CI: Confidence interval
HR: Hazard ratio
IRB: Institutional review board
NMBA: Neuromuscular blocking agents
OR: Odds ratio
PORC: Postoperative residual curarization
SNUBH: Seoul National University Bundang Hospital
