# Supplementary figures and images for: Association of neuromuscular reversal by sugammadex and neostigmine with 90-day mortality after non-cardiac surgery
Source: BMC Anesthesiol. 2020 Feb 20;20:41. doi: 10.1186/s12871-020-00962-7 (PMC7033926; doi:10.1186/s12871-020-00962-7)

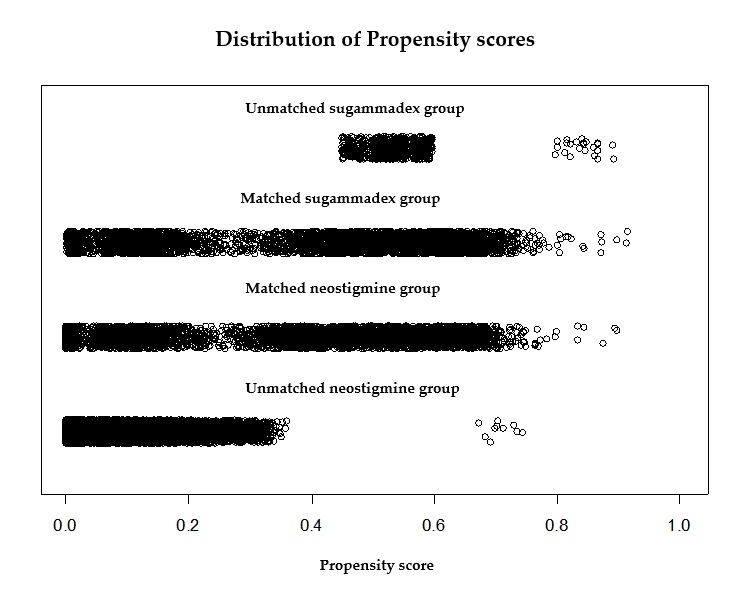

Supplement: Supplementary file 1 — Additional file 1 Figure S1. Distribution of propensity scores before and after propensity score matching [file 12871_2020_962_MOESM1_ESM.jpg]
